# Natural Products as Antimicrobial Agents: From Extraction to Therapeutic Applications

**DOI:** 10.3390/molecules30112393

**Published:** 2025-05-30

**Authors:** Céline Rivière

**Affiliations:** Joint Research Unit 1158 BioEcoAgro, University of Lille, Junia, INRAE, University of Liège, UPJV, University of Artois, ULCO, F-59650 Villeneuve d’Ascq, France; celine.riviere@univ-lille.fr

## 1. Introduction

Antimicrobial resistance, as well as the emergence and re-emergence of some pathogens constitute two major aspects of the Anthropocene epoch [1,2]. The COVID-19 pandemic, caused by severe acute respiratory syndrome coronavirus 2, is a recent example [3]. Globally, the impact of human activities on the environment and on our ability to fight certain pathogens may aggravate this problem. For example, the intensive or inappropriate use of antibiotics, deforestation, and climate change may have significant effects on the emergence of new pathogens and on antimicrobial resistance [4]. In order to counter emerging diseases with a pandemic risk, the WHO promotes the “one health” approach, an integrated and unified approach to human, animal, and environmental health on a global scale [5,6]. New resistance mechanisms are also emerging, making the management of certain diseases very complex and sometimes impossible [1,7]. Therefore, the identification of new antimicrobial agents and the improvement of new therapeutic strategies are urgently required [8]. Natural products, with their immense structural diversity and evolutionary refinement, remain indispensable in the search for therapeutic solutions of infectious diseases (viral, bacterial, fungal, and parasitic) [9,10,11,12,13,14,15,16,17,18,19,20]. This Special Issue aims to disclose the most recent advances in the discovery of antimicrobial agents of natural origin (plant, bacterial, fungal, and animal) in the field of human and veterinary therapy, as well as to describe the new mechanisms of action of some natural antimicrobial agents. This Special Issue is thus dedicated to the exploration of natural products as antimicrobial agents, encompassing investigations covering their extraction, purification, and structural characterization, as well as a mechanistic understanding of their biological effects and therapeutic potential. It includes eight research articles and two review articles, a brief description of which is presented in the following paragraphs.

## 2. An Overview of Published Articles

The study conducted by Lopes et al. (contribution 1) investigated the mucus of *Elysia crispata* Mörch (Placobranchidae), a kleptoplastic marine mollusk, commonly named lettuce sea slug, and its bioactive properties. Their work demonstrated the antimicrobial, antioxidant, and anti-inflammatory potential of the mucus, particularly its proteinaceous and low-molecular-weight fractions. Notably, a strong inhibitory effect against *Pseudomonas aeruginosa*, a critical-priority pathogen according to the WHO, was observed with the mucus native protein extracts. This contribution highlighted the untapped therapeutic promise of marine invertebrates and underscored the importance of marine bioprospecting in antimicrobial discovery.

Mintsa et al. (contribution 2) conducted a comprehensive ethnopharmacological survey among the Fang population of Gabon, then focused their work on the leaves of *Erismadelphus exsul* Mildbr. (Vochysiaceae), a medicinal plant cited in the survey and previously unstudied in phytochemistry. Phytochemical profiling and molecular networking of selected extracts were performed using HPLC-ESI-Q/TOF, which revealed four major families of natural compounds. Significant antibacterial and antioxidant activities were highlighted. This study illustrated the synergy between ethnobotanical fieldwork and modern phytochemistry to confirm the scientific use of these medicinal plants.

Luo et al. (contribution 3) presented a compelling analysis of azalomycin F, a guanidyl-containing macrolide of microbial origin, which targets *Staphylococcus aureus* by disrupting lipoteichoic acid biosynthesis and compromising the bacterial envelope. Their multidisciplinary approach offered detailed mechanistic insights and highlighted the complexity and therapeutic relevance of microbial natural products.

In a complementary study, Chin et al. (contribution 4) examined actinobacterial strains of the family Streptomycetaceae isolated from Singaporean soil. Their chemical and biological analyses led to the discovery of a new compound, tetronomycin A, along with several known antibiotics. The new compound exhibited good antibacterial activity against some *Staphylcoccus aureus* strains. The authors also underlined structure–activity relationships for these different isolated compounds. These results reinforced the central role of actinomycetes in antibiotic discovery and highlighted the importance of bioprospecting diverse ecological niches.

In the field of natural antiviral products, Hakem et al. (contribution 5) screened several *Juncus* species and performed bioguided fractionation on the stems of *Juncus acutus* L. (Juncaceae). They isolated and identified luteolin as an inhibitor of the human coronavirus HCoV-229E. This flavone acted as an inhibitor of the replication step of this coronavirus. Although no significant activity was observed against SARS-CoV-2 or MERS-CoV, the study presented luteolin as a potential compound for the future development of antivirals, particularly against alphacoronaviruses.

The work of Ellatif et al. (contribution 6) evaluated certain biological activities of kefir, including its antimicrobial (antibacterial, antifungal, and antiviral) and wound healing properties in a human gastric epithelial cell model. This work highlighted the antiviral potential of kefir against viral hepatitis C and B. The authors notably demonstrated the anti-HBV efficacy of kefir, supported by its immunomodulatory activity. This study expanded the scope of applications of therapies derived from fermented foods.

A detailed study conducted by Schioppa et al. (contribution 7) explored the stability of different biological-like medias and metabolism of selected C-3 and C-27 triterpene esters that have demonstrated antiparasitic activity in previous works. Metabolic studies performed by a validated HPLC-PDA-HRMS method provided a better understanding of the pharmacokinetic profiles and metabolic fate of these compounds. This work may help in the development of formulations that improve oral bioavailability.

In the context of bacterial resistance, Oo et al. (contribution 8) examined extracts and essential oils of *Myristica fragrans* Houtt. (Myristicaceae) seeds for their ability to inhibit efflux pumps in methicillin-resistant *Staphylococcus aureus* (MRSA). Their results suggested that these natural products can potentiate ciprofloxacin activity by targeting resistance mechanisms, an increasingly important strategy in antimicrobial therapeutics.

This Special Issue also features two review articles. The first, by Al-shaibani et al. (contribution 9), provided a comprehensive overview of the specialized metabolites of the phylum Actinobacteria and their therapeutic applications. The authors highlighted the adaptive biosynthetic capabilities of these organisms in extreme environments, reinforcing their importance as a reservoir of novel antibiotics. The second, by Ratajczak et al. (contribution 10), examined the antimicrobial potential of bee products—honey, propolis, royal jelly, and bee venom—while addressing the challenges of standardization and clinical application of these products.

Collectively, the contributions in this Special Issue highlight the continued importance of natural product research in the development of antimicrobial agents. From traditional medicinal plants to marine and microbial sources, the scope for bioactive compound discovery remains vast. Moreover, the integration of ethnopharmacology, advanced analytical tools, and molecular biology provides a deeper understanding of the bioactivity and therapeutic potential of these products.

## 3. Conclusions

In conclusion, this compilation of original research and reviews highlights the innovation and multidisciplinary efforts that are shaping the future of antimicrobial therapy through natural products. As Editor, I extend my sincere gratitude to the authors, reviewers, and editorial team for their valuable contributions. I hope this Special Issue serves as a catalyst for further exploration and collaboration in the field of natural product-based antimicrobial research, and that it inspires continued scientific pursuit in addressing global health challenges.
